# Metallothermic Reduction of MoO_3_ on Combustion Synthesis of Molybdenum Silicides/MgAl_2_O_4_ Composites

**DOI:** 10.3390/ma14174800

**Published:** 2021-08-24

**Authors:** Chun-Liang Yeh, Min-Chia Chen

**Affiliations:** Department of Aerospace and Systems Engineering, Feng Chia University, Taichung 40724, Taiwan; m0825504@fcu.edu.tw

**Keywords:** molybdenum silicides, MgAl_2_O_4_, aluminothermic, magnesiothermic, self-propagating high-temperature synthesis

## Abstract

Combustion synthesis involving metallothermic reduction of MoO_3_ by dual reductants, Mg and Al, to enhance the reaction exothermicity was applied for the in situ production of Mo_3_Si–, Mo_5_Si_3_− and MoSi_2_–MgAl_2_O_4_ composites with a broad compositional range. Reduction of MoO_3_ by Mg and Al is highly exothermic and produces MgO and Al_2_O_3_ as precursors of MgAl_2_O_4_. Molybdenum silicides are synthesized from the reactions of Si with both reduced and elemental Mo. Experimental evidence indicated that the reaction proceeded as self-propagating high-temperature synthesis (SHS) and the increase in silicide content weakened the exothermicity of the overall reaction, and therefore, lowered combustion front temperature and velocity. The XRD analysis indicated that Mo_3_Si–, Mo_5_Si_3_– and MoSi_2_–MgAl_2_O_4_ composites were well produced with only trivial amounts of secondary silicides. Based on SEM and EDS examinations, the morphology of synthesized composites exhibited dense and connecting MgAl_2_O_4_ crystals and micro-sized silicide particles, which were distributed over or embedded in the large MgAl_2_O_4_ crystals.

## 1. Introduction

Molybdenum silicides, Mo_3_Si, Mo_5_Si_3_ and MoSi_2_, are promising intermetallic materials for ultrahigh-temperature structural applications. Besides a high melting point over 2020 °C, they possess high strength, excellent oxidation resistance, corrosion resistance, creep resistance and good compatibility with ceramic reinforcements [1,2,3,4,5,6,7,8]. To improve the refractory property of transition metal silicides, magnesium aluminate spinel (MgAl_2_O_4_) has been one of the potential additives, because of its unique combination of properties, including a high melting point (2135 °C), relatively low density, chemical inertness, high hardness, high mechanical strength and good thermal shock resistance [9,10,11,12]. However, preparation of MgAl_2_O_4_ via either wet chemical methods or solid-state reactions required several complicated steps under the long processing time [9,10,11,12]. 

As an alternative, metallothermic reduction reactions (MRRs) of metal oxides with Mg and Al as reducing agents produce MgO and Al_2_O_3_ as precursors for the formation of MgAl_2_O_4_ and such oxidation reactions are highly exothermic [13,14]. When combining Mg/Al-based MRRs with combustion synthesis, such a fabrication route is effective in producing MgAl_2_O_4_-containing composites. Moreover, the highly-exothermic MRRs render reduction-based combustion synthesis fit for self-propagating high-temperature synthesis (SHS). Many merits such as high energy efficiency, short reaction time, simplicity of operation and high-purity products have been recognized for the SHS process [15,16,17]. According to Horvitz and Gotman [18], reduction-based combustion synthesis using 2TiO_2_–Mg–4Al samples was performed to produce TiAl–Ti_3_Al–MgAl_2_O_4_ composites. Omran et al. [19] conducted co-reduction of WO_3_ and B_2_O_3_ by Mg in the presence of Al_2_O_3_ to fabricate the composites of MgAl_2_O_4_–W–W_2_B. By means of adopting pre-added MgO, Zaki et al. [20] obtained MgAl_2_O_4_ composites with MoSi_2_ and Mo_5_Si_3_ from co-reduction of SiO_2_ and MoO_3_ by Al in argon at a pressure of 5 MPa. The high Ar pressure was to suppress the volatilization of MoO_3_. Recently, Radishevskaya et al. [21] synthesized MgAl_2_O_4_ by the SHS method using the reactant mixtures consisting of MgO and Al_2_O_3_, along with Al as the fuel, Mg(NO_3_)_2_⋅H_2_O as the oxidizer, and NaCl as the mineralizer. Results indicated that NaCl of 1 wt.% contributed to the completion of the formation of MgAl_2_O_4_ and mechanical activation of the green mixture for 60 s facilitated the production of MgAl_2_O_4_ without oxide impurities. 

By using Mg and Al simultaneously as dual reductants, this work aims at investigating the in situ production of MgAl_2_O_4_-containing molybdenum silicide (Mo_3_Si, Mo_5_Si_3_, and MoSi_2_) composites by the SHS process with reducing stages. That is, a solid-state combustion reaction involves the synthesis of MgAl_2_O_4_ from the metallothermic reduction of MoO_3_ and the formation of molybdenum silicides from elemental interactions between Mo and Si. Three different silicide phases were produced and their influence on reaction exothermicity and combustion wave kinetics was explored. Compositional and microstructural analyses were performed on the final composites. Moreover, some products were selected for Vickers hardness and fracture toughness measurements. 

## 2. Materials and Methods

The raw materials utilized by this study include MoO_3_ (Acros Organics, 99.5%), Mg (Alfa Aesar, <45 μm, 99.8%), Al (Showa Chemical Co., <45 μm, 99.9%), Mo (Strem Chemicals, <45 μm, 99.9%), Si (Strem Chemicals, <45 μm, 99.5%), and Al_2_O_3_ (Alfa Aesar, 99%). According to different molybdenum silicides, three reaction systems, R(1), R(2), and R(3), are formulated for the synthesis of Mo_3_Si–, Mo_5_Si_3_–, and MoSi_2_–MgAl_2_O_4_ composites, respectively.

(1) $$\frac{4}{3}MoO_{3}+Mg+2Al+\left(3x-\frac{4}{3}\right)Mo+xSi\to xMo_{3}Si+MgAl_{2}O_{4}$$

(2) $$\frac{4}{3}MoO_{3}+Mg+2Al+\left(5y-\frac{4}{3}\right)Mo+3ySi\to yMo_{5}Si_{3}+MgAl_{2}O_{4}$$

(3) $$\frac{13}{12}MoO_{3}+Mg+\frac{3}{2}Al+\frac{1}{4}Al_{2}O_{3}+\left(z-\frac{13}{12}\right)Mo+2zSi\to zMoSi_{2}+MgAl_{2}O_{4}$$

where stoichiometric coefficients *x*, *y*, and *z* are associated with the quantities of Mo and Si powders in the green mixtures, and also represent the molar proportion of silicide phase to MgAl_2_O_4_. The same composition of metallothermic reagents of 4/3MoO_3_ + Mg + 2Al is adopted in R(1) and R(2), but R(3) has a different metallothermic mixture of 13/12MoO_3_ + Mg + 3/2Al because R(3) comprises pre-added Al_2_O_3_. Because of metallothermic reduction of MoO_3_, the source of Mo for the formation of molybdenum silicides (Mo_3_Si, Mo_5_Si_3_, and MoSi_2_) from R(1), R(2), and R(3) included both reduced and elemental Mo. 

It has been realized that magnesiothermic and aluminothermic reductions of MoO_3_ are highly exothermic and have an adiabatic temperature (*T*_ad_) exceeding 4200 K [22], which plays an important role in facilitating self-sustaining combustion for R(1), R(2) and R(3). When compared with the reduction of MoO_3_ by Mg and Al, the formation reactions of Mo_3_Si, Mo_5_Si_3_ and MoSi_2_ are much less energetic. Among three molybdenum silicides, MoSi_2_ is the most exothermic phase to form [23], and therefore, Al_2_O_3_ at one-quarter of the required amount was added in the starting mixture to regulate the degree of violence of combustion. 

Experimental ranges of *x*, *y*, and *z* conducted in this study were determined based on the reaction exothermicity of R(1), R(2) and R(3), which was assessed by computing *T*_ad_ as a function of stoichiometric coefficients according to the following energy balance equation [24,25] with thermochemical data taken from [23].

(4) $$\Delta H_{r}+\int_{298}^{T_{ad}}\sum^{\;}n_{j}C_{p}\left(P_{j}\right)dT+\sum_{298-T_{ad}}n_{j}L\left(P_{j}\right)=0$$

where Δ*H_r_* is the enthalpy of reaction at 298 K, *n*_j_ is the stoichiometric coefficient, *C_p_* and *L* are the specific heat and latent heat, respectively, and *P_j_* refers to the product. 

The value of Δ*H_r_* was calculated from the difference in enthalpy of formation (Δ*H_f_*) between the reactants (Δ*H_f_* of MoO_3_: −745 kJ/mol, Al_2_O_3_: –1675.7 kJ/mol, and Mg, Al, Mo, and Si: 0 kJ/mol) and products (Δ*H_f_* of Mo_3_Si: −118.4 kJ/mol, Mo_5_Si_3_: −310.6 kJ/mol, MoSi_2_: −131.4 kJ/mol, and MgAl_2_O_4_: −2299.1 kJ/mol) [23]. The values of *C_p_* of the products as a function of temperature are expressed as follows [23].

(5) $$C_{p}\left(Mo_{3}Si\right)=85.23+22.68\times 10^{-3}\times T+0.03\times 10^{6}\times T^{-2}\;(J\cdot {\mathrm{mol}}^{-1}\cdot K^{-1})$$

(6) $$C_{p}\left(Mo_{5}Si_{3}\right)=183.36+35.01\times 10^{-3}\times T-1.2\times 10^{6}\times T^{-2}\;(J\cdot {\mathrm{mol}}^{-1}\cdot K^{-1})$$

(7) $$C_{p}\left(MoSi_{2}\right)=67.84+11.95\times 10^{-3}\times T-0.66\times 10^{6}\times T^{-2}\;(J\cdot {\mathrm{mol}}^{-1}\cdot K^{-1})$$

(8) $$C_{p}\left(MgAl_{2}O_{4}\right)=146.78+35.56\times 10^{-3}\times T-3.68\times 10^{6}\times T^{-2}\;(J\cdot {\mathrm{mol}}^{-1}\cdot K^{-1})$$

The SHS experiment was performed in a windowed combustion chamber filled with high-purity argon (99.99%) at 0.2 MPa. Reactant powders were dry mixed and then were uniaxially pressed to form cylindrical test specimens with 12 mm in height, 7 mm in diameter, and 55% in the relative density. In this work, a cylindrical bottle partially filled with the raw materials and alumina (Al_2_O_3_) grinding balls rotated about the longitudinal axis of a tumbler ball mill machine for 8 h to fully blend the reactant powders. The size of the alumina ball is 5 mm in diameter. The ball mill operated at 90 rpm. Because Al_2_O_3_ is one of the precursors to form MgAl_2_O_4_, no contamination from grinding balls was detected. 

The combustion wave propagation velocity (*V*_f_) was determined from the time series of recorded combustion videos. The combustion temperature was measured by a 125 μm bead-sized thermocouple with an alloy composition of Pt/Pt–13%Rh. Details of the experimental setup were previously reported [25,26]. Phase components of the synthesized products were identified by an X-ray diffractometer with CuK_α_ radiation (Bruker D2 Phaser, Billerica, MA, USA). Analyses of scanning electron microscopy (SEM) (Hitachi S3000H, Tokyo, Japan) and energy dispersive spectroscopy (EDS) were performed to examine the fracture surface microstructure and composition ratio of elements of the final products. 

Measurement of Vickers hardness and fracture toughness of the products was performed [27]. For such measurements, only selected experiments under stoichiometric coefficients of *x* = *y* = *z* = 2 were carried out by placing the sample compact in a stainless-steel mold. Densification of the product was conducted by a hydraulic compressor. Upon the completion of the SHS reaction, the burned sample was rapidly pressed when the product was still hot and plastic, which was held for about 15 s. The product density after compression reached about 93–95% of theoretical density and then the product surface was polished for the measurement. Microhardness was measured with a Buehler Micromet microhardness tester at a load of 1000 g and a dwelling time of 10 s. Five indentations were made to obtain the average values of the indentation imprint and crack length measurements. 

In this study, Vickers hardness (*H*_v_) was calculated from the applied load (*P*) and the average diagonal impression length (*d*) in the equation below [28,29]. The fracture toughness (*K*_IC_) was determined by the indentation method using the following equation proposed by Evans and Charles [29].

(9) $$H_{v}=1.8544\frac{P}{d^{2}}$$

(10) $$K_{IC}=0.16H_{v}a^{1/2}{\left(\frac{c}{a}\right)}^{-3/2}$$

where *a* is the half of the average length of two diagonals of the indentation and *c* the radial crack length measured from the center of the indentation. 

## 3. Results and Discussion

### 3.1. Combustion Exothermicity of Reactions

**Figure 1:**
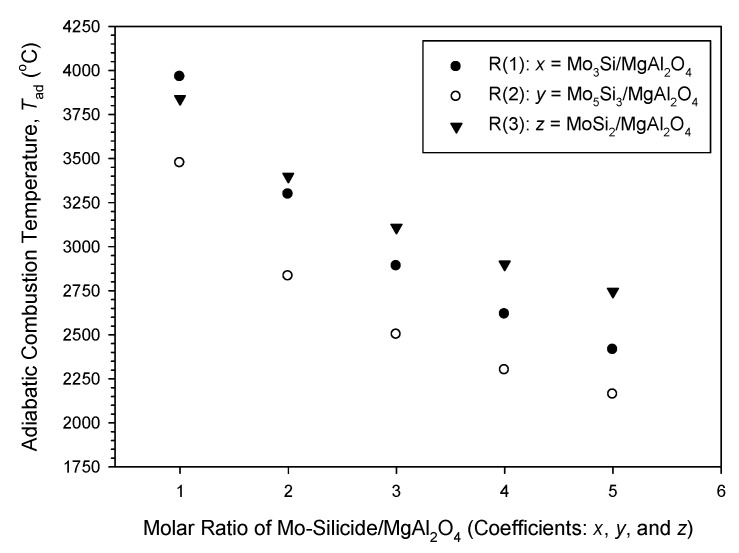
Variations of adiabatic combustion temperatures (*T*_ad_) with molar ratios of Mo-silicide/ MgAl_2_O_4_ of products synthesized from R(1), R(2), and R(3).

Calculated values of *T*_ad_ of R(1), R(2) and R(3) as a function of their respective stoichiometric coefficients are presented in Figure 1 in order to evaluate combustion exothermicity. A significant decrease in *T*_ad_ with increasing silicide content is observed for all three synthesis reactions, mainly because the formation of molybdenum silicides is much less exothermic than the metallothermic reduction of MoO_3_. As revealed in Figure 1, the value of *T*_ad_ associated with the formation of Mo_3_Si–MgAl_2_O_4_ composites from R(1) decreases considerably from 3964 °C to 2415 °C as the coefficient *x* increases from 1 to 5. On account of a large heat capacity for Mo_5_Si_3_, R(2) is the weakest exothermic reaction and shows a decrease in *T*_ad_ from 3475 °C at *y* = 1 to 2162 °C at *y* = 5. In spite of the dilution effect of pre-added Al_2_O_3_ on combustion, R(3) intended for the synthesis of MoSi_2_–MgAl_2_O_4_ composites is still very energetic with *T*_ad_ ranging from 3840 °C to 2745 °C. Figure 1 indicates that R(3) has the highest *T*_ad_ except for the case of *z* = 1. According to the analysis of combustion exothermicity, R(1) and R(3) were conducted in this study with the experimental variables of *x* = 2–5 and *z* = 2–5, respectively, and R(2) with *y* = 1–4 was carried out. Reactions with *x* = 1 and *z* = 1 were avoided, since the resulting combustion was often violent enough to melt down the powder compact and led to incomplete phase conversion. 

### 3.2. Combustion Wave Velocity and Temperature

**Figure 2:**
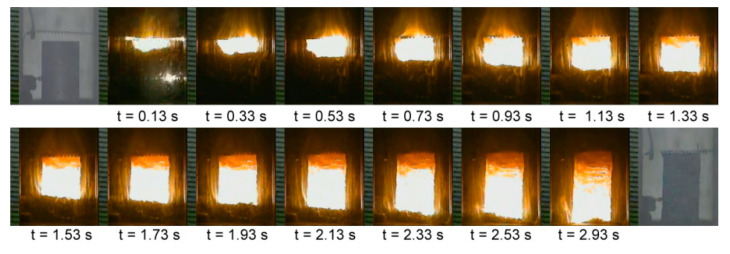
A typical sequence of self-sustaining combustion images recorded from R(1) with *x* = 3.

**Figure 3:**
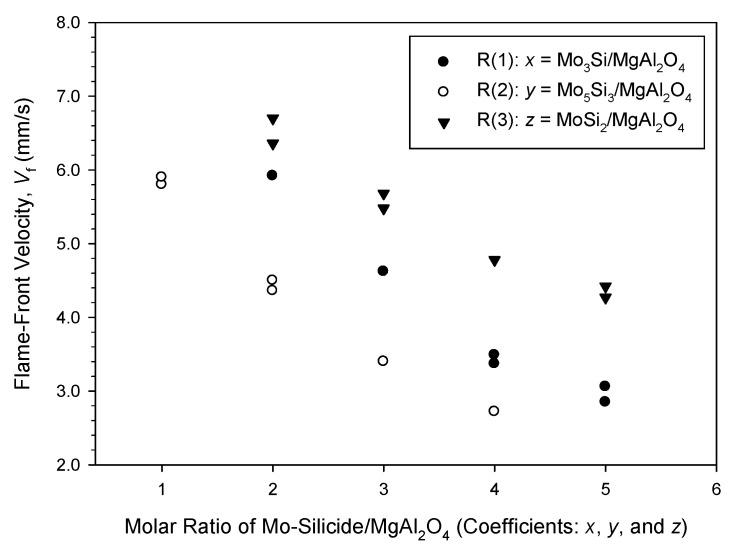
Variations of flame-front propagation velocities with stoichiometric coefficients (*x*, *y*, and *z*) of R(1), R(2), and R(3).

A typical sequence of recorded combustion images from R(1) with *x* = 3 is illustrated in Figure 2, showing a stable and self-sustaining combustion process. A distinct combustion front allowed the propagation velocity to be determined. Variations of combustion wave velocities of R(1), R(2) and R(3) with the molar ratio of silicide to MgAl_2_O_4_ are presented in Figure 3. A declining trend consistent with the adiabatic combustion temperature was observed. This can be explained by the fact that the combustion wave propagation rate is essentially governed by layer-by-layer heat transfer from the thin combustion zone to the unreacted region, and therefore, is subject to the reaction front temperature. Specifically, Figure 3 points out a decrease in *V*_f_ from 5.9 to 2.9 mm/s for R(1) with *x* from 2 to 5. For the similar range of stoichiometry of *z* = 2–5, R(3) has a faster combustion wave with *V*_f_ ranging from 6.7 to 4.3 mm/s. On the other hand, the combustion front of R(2) has a slower speed and its *V*_f_ decreases from 5.9 mm/s at *y* = 1 to 2.7 mm/s at *y* = 4. 

**Figure 4:**
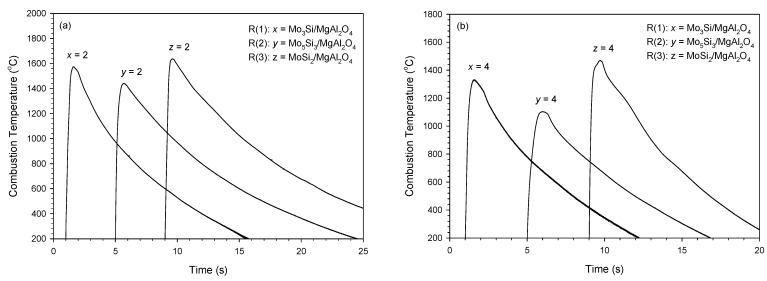
Combustion temperature profiles of R(1), R(2), and R(3) with stoichiometric coefficients of (**a**) *x* = *y* = *z* = 2 and (**b**) *x* = *y* = *z* = 4.

Figure 4a,b depict combustion temperature profiles measured from R(1), R(2) and R(3) under equal stoichiometric coefficients of 2 and 4, respectively. A steep rising gradient followed by a rapid cooling rate is characteristic of the temperature profile of the SHS reaction. The highest value is considered as the combustion front temperature (*T*_c_). A comparison of *T*_c_ among three synthesis reactions in Figure 4a indicates that R(3) has the highest *T*_c_ of 1637 °C (*z* = 2), R(2) has the lowest 1442 °C (*y* = 2), and R(1) is in-between at 1574 °C (*x* = 2). A similar ranking of *T*_c_ can be seen in Figure 4b, which is associated with the synthesis of composites with a molar ratio silicide/MgAl_2_O_4_ equal to 4. When compared with *T*_c_ shown in Figure 4a, lower values of *T*_c_ = 1330 °C, 1103 °C and 1470 °C are observed in Figure 4b for R(1), R(2) and R(3), respectively. This confirms the decrease in reaction exothermicity with an increasing fraction of silicide formed in the composite.

### 3.3. Composition and Microstructure Analyses of SHS-Derived Products

**Figure 5:**
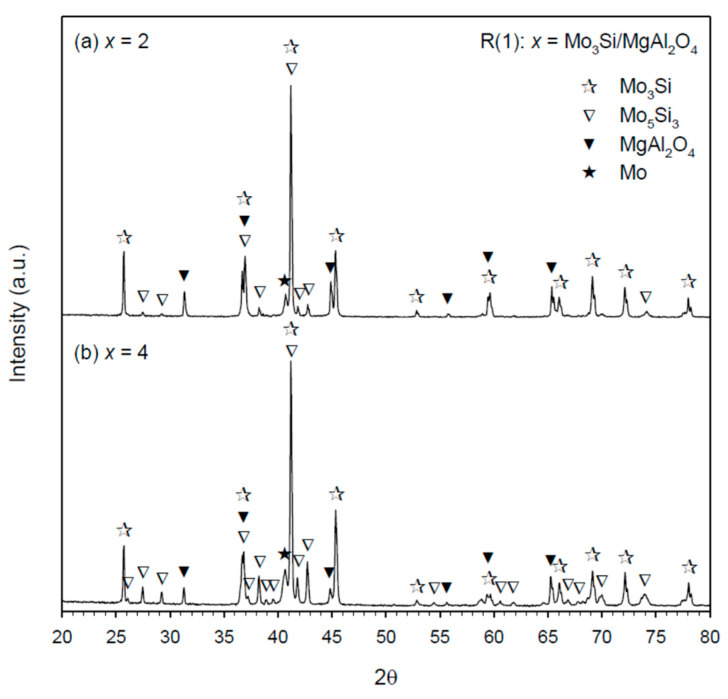
XRD patterns of as-synthesized Mo_3_Si–MgAl_2_O_4_ composites from R(1) with (**a**) *x* = 2 and (**b**) *x* = 4.

**Figure 6:**
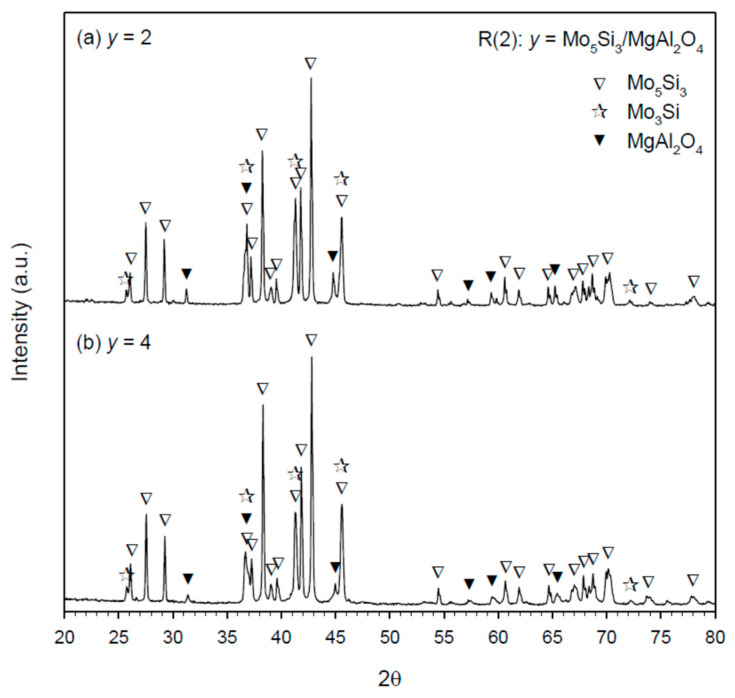
XRD patterns of as-synthesized Mo_5_Si_3_–MgAl_2_O_4_ composites from R(2) with (**a**) *y* = 2 and (**b**) *y* = 4.

The XRD spectrum graphs of final products synthesized from R(1) with *x* = 2 and 4 are plotted in Figure 5a,b, respectively. Besides MgAl_2_O_4_, two silicide compounds were detected with Mo_3_Si the dominant and Mo_5_Si_3_ the minor. Because of the presence of Mo_5_Si_3_, there was a small amount of elemental Mo left in the end product. It should be noted that the production of MgAl_2_O_4_ justifies a combination reaction between in situ formed Al_2_O_3_ and MgO from the metallothermic reduction of MoO_3_ by dual reductants. Phase constituents associated with the products of R(2) are identified in Figure 6a,b, indicative of the Mo_5_Si_3_–MgAl_2_O_4_ composites with a trivial amount of Mo_3_Si. Because Mo_5_Si_3_ has a homogeneity range from 37.5 to 40 at% Si [20], no remnant Si was found in the Mo_5_Si_3_–MgAl_2_O_4_ products even containing some Mo_3_Si. 

**Figure 7:**
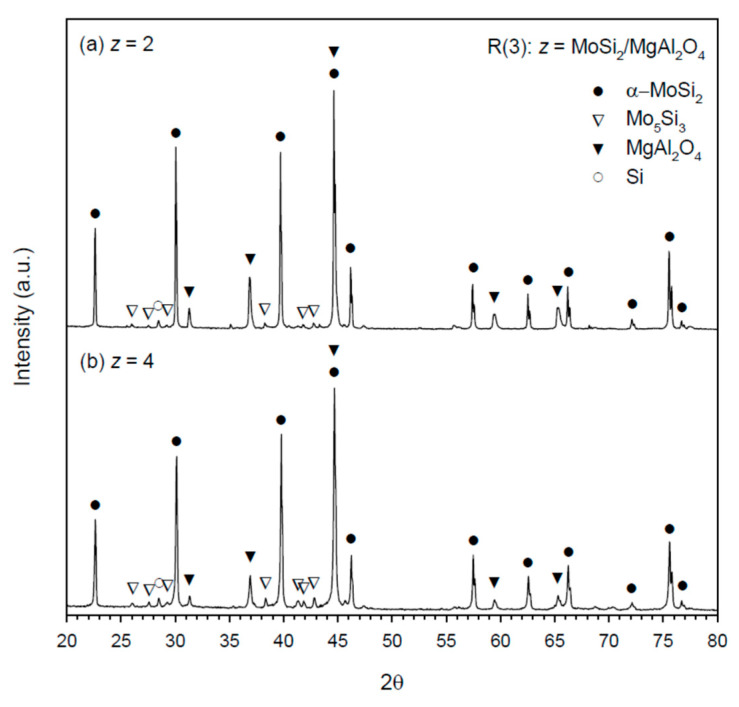
XRD patterns of as-synthesized MoSi_2_–MgAl_2_O_4_ composites from R(3) with (**a**) *z* = 2 and (**b**) *z* = 4.

Figure 7a,b shows the XRD spectra of the MoSi_2_–MgAl_2_O_4_ composites produced from R(3) with *z* = 2 and 4, respectively. It should be pointed out that MoSi_2_ formed from R(3) is α-MoSi_2_ (the low-temperature phase). This was due to the fact that the reaction temperature of R(3) was below 1900 °C [22], the phase transition temperature from α-MoSi_2_ to the high-temperature phase of β-MoSi_2_. As revealed in Figure 7a,b, there are small amounts of Mo_5_Si_3_ and Si in the as-synthesized MoSi_2_–MgAl_2_O_4_ composites. 

When compared with the work of Zaki et al. [20], MgAl_2_O_4_ composites with MoSi_2_ and Mo_5_Si_3_ were produced from MoO_3_, SiO_2_, Al and MgO powder mixtures by self-sustaining combustion. They indicated the presence of small amounts of Mo_5_Si_3_, Al_2_SiO_5_ and free Si in the synthesized MgAl_2_O_4_–MoSi_2_ composites. The impurity Al_2_SiO_5_ was formed via a combination reaction of Al_2_O_3_ with SiO_2_. Moreover, the increase in MgO led to the formation of the other impurity Mg_2_SiO_4_ which was produced from the reaction between MgO and SiO_2_. Therefore, it is believed that the formation of Al_2_SiO_5_ and Mg_2_SiO_4_ could be due to incomplete reduction of SiO_2_, since these two phases were not found in the products of the present study. On the other hand, Zaki et al. [20] obtained MgAl_2_O_4_–Mo_5_Si_3_ composites without impurities and secondary silicides, on account of a larger heat release from combustion and a lesser amount of SiO_2_ contained in the sample. 

In the work of Radishevskaya et al. [21], MgO and Al_2_O_3_ were added into a combustible mixture composed of Al, Mg(NO_3_)_2_⋅H_2_O and NaCl to produce MgAl_2_O_4_ through the SHS scheme. Results showed that the pre-added MgO and Al_2_O_3_ failed to be fully combined into MgAl_2_O_4_ unless mechanical activation of initial components in a planetary mill was conducted. In contrast, MgO and Al_2_O_3_ were not detected in the final composites of R(1), R(2) and R(3). This could be because these two precursors of MgAl_2_O_4_ were in situ produced from metallothermic reduction reactions in the present study.

**Figure 8:**
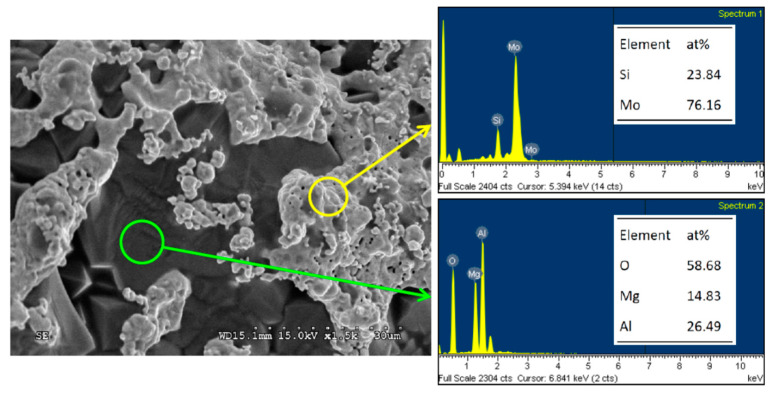
SEM image and EDS element spectra of Mo_3_Si–MgAl_2_O_4_ composite synthesized from R(1) with *x* = 3.

For the Mo_3_Si–MgAl_2_O_4_ composite of R(1) with *x* = 3 illustrated in Figure 8, the SEM image shows the fracture surface microstructure and EDS spectra provide the atomic ratios of constitution elements. The micrograph exhibits that MgAl_2_O_4_ crystals are dense and continuous and small Mo_3_Si grains tend to agglomerate into clusters. Moreover, the atomic ratios of Mo:Si = 76.16:23.84 and Mg:Al:O = 14.83:26.49:58.68 are close to those of Mo_3_Si and MgAl_2_O_4_. 

**Figure 9:**
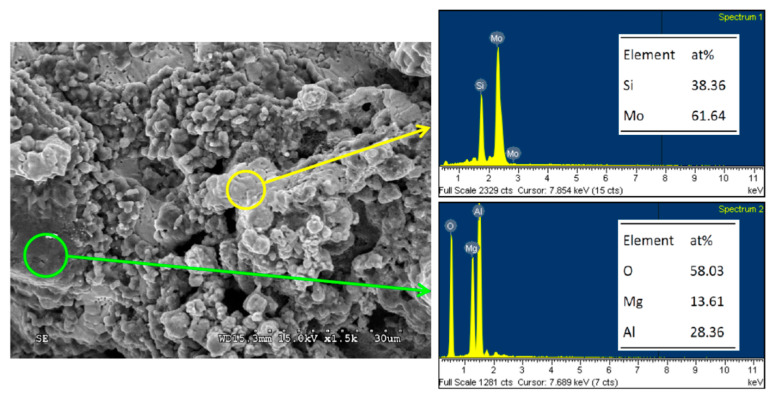
SEM image with EDS element spectra of Mo_5_Si_3_–MgAl_2_O_4_ composite synthesized from R(2) with *y* = 3.

**Figure 10:**
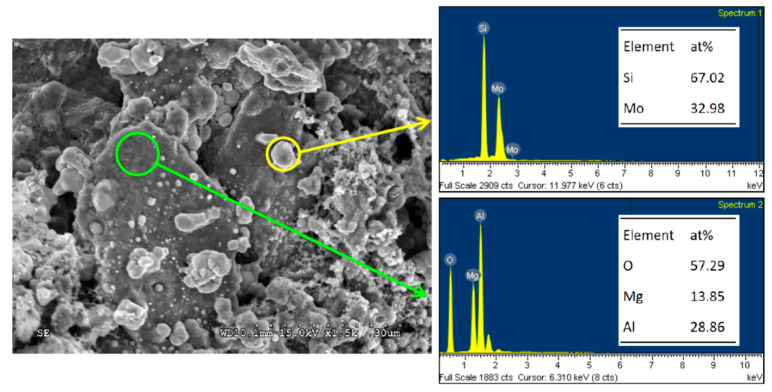
SEM image with EDS element spectra of MoSi_2_–MgAl_2_O_4_ composite synthesized from R(3) with *z* = 3.

The microstructure of the Mo_5_Si_3_–MgAl_2_O_4_ composite of R(2) with *y* = 3 in Figure 9 also reveals agglomeration of small Mo_5_Si_3_ grains with a particle size of about 2–4 μm. Most of the large MgAl_2_O_4_ crystals are covered with Mo_5_Si_3_ grains. The atomic ratios of Mo:Si = 61.64:38.36 and Mg:Al:O = 13.61:28.36:58.03 confirm the formation of Mo_5_Si_3_ and MgAl_2_O_4_. A similar morphology can be seen in Figure 10, unveiling the MoSi_2_–MgAl_2_O_4_ composite of R(3) with *z* = 3. It is evident that MgAl_2_O_4_ crystals are dense and relatively large. Small MoSi_2_ grains are distributed over or embedded in MgAl_2_O_4_ crystals. Atomic ratios of Mo:Si = 32.98:67.02 and Mg:Al:O = 13.85:28.86:57.29 were obtained from the EDS analysis. 

Selected test conditions (*x* = *y* = *z* = 2) were conducted to prepare product samples for the measurement of hardness and fracture toughness. For the composite of 2Mo_3_Si–MgAl_2_O_4_ produced from R(1), Vickers hardness of *H*_v_ = 1.41 × 10^4^ MPa and fracture toughness of *K*_IC_ = 3.3 MPa m^1/2^ were determined. Values of *H*_v_ = 1.42 × 10^4^ MPa and *K*_IC_ = 3.1 MPa m^1/2^ were obtained for 2Mo_5_Si_3_–MgAl_2_O_4_ synthesized from R(2). For the product of 2MoSi_2_–MgAl_2_O_4_ from R(3), *H*_v_ = 1.48 × 10^4^ MPa and *K*_IC_ = 2.8 MPa m^1/2^ were determined. The error of hardness values was estimated as about ±10% and the error of fracture toughness values was within ±20%. The uncertainty of *K*_IC_ determination using the indentation fracture method could result from the residual stresses induced by specimen densification, the existence of pores or cracks, surface finish, and possible inhomogeneous microstructure. Compared with monolithic Mo_3_Si, Mo_5_Si_3_, and MoSi_2_ (*H*_v_ ≈ 1.3 × 10^4^ MPa and *K*_IC_ = 2~3 MPa m^1/2^) [7,30,31], MgAl_2_O_4_ as an additive improved the hardness and toughness of molybdenum silicides. 

## 4. Conclusions

The in situ fabrication of Mo_3_Si–, Mo_5_Si_3_– and MoSi_2_–MgAl_2_O_4_ composites was investigated by the SHS process integrating metallothermic reduction of MoO_3_ with combustion synthesis. Mg and Al were simultaneously used as dual reductants to produce MgO and Al_2_O_3_ as precursors of MgAl_2_O_4_. Molybdenum silicides were synthesized from the elemental reactions between Mo and Si. Experimental results showed that the formation of MoSi_2_–MgAl_2_O_4_ composites was the most exothermic and characterized by the highest combustion front temperature and fastest combustion velocity, while that of Mo_5_Si_3_–MgAl_2_O_4_ composites was the least. Composites with molar ratios of Mo_3_Si/MgAl_2_O_4_ from 2 to 5, Mo_5_Si_3_/MgAl_2_O_4_ from 1 to 4, and MoSi_2_/MgAl_2_O_4_ from 2 to 5 were synthesized. An increase in silicide content brought about a decrease in reaction exothermicity because the formation of molybdenum silicides was much less exothermic than the metallothermic reduction of MoO_3_. Based on the XRD patterns, phase conversion from the reactants to products was essentially completed except for trivial amounts of secondary silicide and Mo or Si present in the end products. SEM and EDS analyses revealed that MgAl_2_O_4_ formed large connecting grains with a dense morphology. Granular Mo_3_Si, Mo_5_Si_3_ and MoSi_2_ were relatively small and were distributed over or embedded in MgAl_2_O_4_ crystals. Hardness and fracture toughness of molybdenum silicides were improved by adding MgAl_2_O_4_. This study demonstrated an effective fabrication route adopting dual reductants to increase combustion exothermicity for the in situ production of Mo_3_Si–, Mo_5_Si_3_– and MoSi_2_–MgAl_2_O_4_ composites with a broad compositional range. 

## Data Availability

Data presented in this study are available in the article.
